# Higher magnesium depletion score increases the risk of all‑cause and cardiovascular mortality in US adults with diabetes

**DOI:** 10.1371/journal.pone.0314298

**Published:** 2025-01-20

**Authors:** Hao Zhang, Liping Kuang, Qiang Wan

**Affiliations:** 1 Graduate School of Jiangxi University of Chinese Medicine, Nanchang, Jiangxi, China; 2 Jiangxi University of Chinese Medicine, Nanchang, Jiangxi, China; 3 Department of Cardiology, Affiliated Hospital of Jiangxi University of Chinese Medicine, Nanchang, Jiangxi, China; Tehran University of Medical Sciences, ISLAMIC REPUBLIC OF IRAN

## Abstract

**Background:**

Both dietary magnesium and serum magnesium are associated with the prognosis of diabetic patients. However, the impact of the magnesium depletion score (MDS), which assesses systemic magnesium deficiency, on the prognosis of diabetic patients remains unclear. This cohort study aims to explore the potential association between the MDS and all-cause and cardiovascular mortality in diabetic patients.

**Methods:**

In this study, we analyzed data from 5,219 diabetic individuals from National Health and Nutrition Examination Survey (NHANES) 2003–2018. Participant mortality information was sourced from the National Death Index records. MDS was divided into lower MDS (0–1 points), middle MDS (2 points), and higher MDS (3–5 points) groups. Weighted multivariable Cox regression was utilized to explore the potential association between MDS and mortality in diabetic patients. Stratified analyses and sensitivity analyses were employed to validate the robustness of our findings.

**Results:**

Among the 5,219 participants included in this study, 1,212 experienced all-cause mortality, and 348 experienced cardiovascular mortality. Weighted multivariable Cox regression indicated that higher MDS was strongly linked to a heightened risk of mortality in all models, including the fully adjusted model (all-cause mortality: HR = 1.58, 95% CI: 1.20–2.08; cardiovascular mortality: HR = 1.92, 95% CI: 1.28–2.88). In the stratified analysis, we found that the association between MDS and all-cause mortality was stronger among individuals aged <60 years. No significant differences were found in the relationship between MDS and mortality within other subgroups. In the sensitivity analyses, our results remained robust.

**Conclusions:**

An increase in MDS is significantly correlated with a higher risk of all-cause and cardiovascular mortality in diabetic patients. The risk of all-cause mortality was higher in diabetic patients aged <60. Early monitoring and management of MDS, as well as optimizing magnesium nutritional status, may benefit diabetic patients.

## Introduction

Diabetes has posed a significant global health burden for the last thirty years [1, 2]. As reported in the Global Burden of Disease Study 2021, approximately 529 million people currently live with diabetes globally, and this figure is expected to surpass 1.31 billion by 2050 [3]. Previous studies have indicated that diabetes elevates the risk of multiple conditions, such as cardiovascular disease, chronic kidney disease, and neuropathy [4–6]. Furthermore, the present risks of overall mortality and heart-related death remain elevated for those with diabetes [7, 8]. Therefore, timely identification of additional risk factors and early intervention are crucial for reducing the mortality risk associated with diabetes.

Magnesium is a crucial mineral in the human body, involved in almost all critical metabolic and biochemical processes within cells [9]. It is fundamental in regulating glucose homeostasis and maintaining normal blood sugar levels [10]. Research indicates that hypomagnesemia is associated with an increased risk of adverse cardiovascular diseases in patients with type 2 diabetes [11]. Increasing dietary magnesium intake may reduce the risk of diabetes and overall mortality [12]. Previous studies have primarily concentrated on the effects of serum magnesium and dietary magnesium on the prognosis of diabetic patients, while research on the implications of magnesium deficiency for their prognosis remains limited.

Recently, Fan et al. developed the magnesium depletion score (MDS), a novel clinical indicator for assessing systemic magnesium deficiency [13]. It considers four common risk factors in the United States that significantly affect renal magnesium reabsorption, including the current use of diuretics and PPIs, heavy alcohol consumption, and kidney disease [13–16]. Moreover, MDS has been shown to have superior predictive performance for magnesium deficiency compared to serum magnesium and urinary magnesium levels. Higher MDS levels indicate a more severe degree of magnesium deficiency, which may offer new insights for identifying magnesium-deficient patients, optimizing magnesium nutritional status, and improving clinical outcomes. To our knowledge, there is currently only one study that has explored the relationship between MDS and the risk of diabetes [17]. The potential association between MDS and the prognosis of diabetic patients remains to be explored.

Given the limited research exploring the relationship between magnesium deficiency and the prognosis of diabetic patients, our study aims to investigate the potential link between MDS and mortality in this population. We utilized data from National Health and Nutrition Examination Survey (NHANES) 2003–2018 participants to accomplish this objective.

## Methods

### Data sources

The National Center for Health Statistics (NCHS) conducts the NHANES survey program, which is intended to evaluate the health and nutritional status of both adults and children in the United States [18]. It collects participants’ health data through a combination of interviews and physical assessments, with interviews carried out using questionnaires and physical examinations conducted by trained medical professionals. The interviews primarily collect information on sociodemographic characteristics, diet, and health-related questions, while the physical examinations include laboratory tests and physiological measurements. The survey uses a complex, stratified, multistage probability sampling method to better represent the non-institutionalized U.S. resident population. The Ethics Review Board of the NCHS approved the NHANES study protocol, and all participants gave written informed consent [19]. Data collection and analysis for this study were completed between April and July 2024. All data from NHANES participants are publicly available and can be accessed for free on the NHANES website (https://www.cdc.gov/nchs/nhanes/index.htm).

### Study design and population

**Fig 1:**
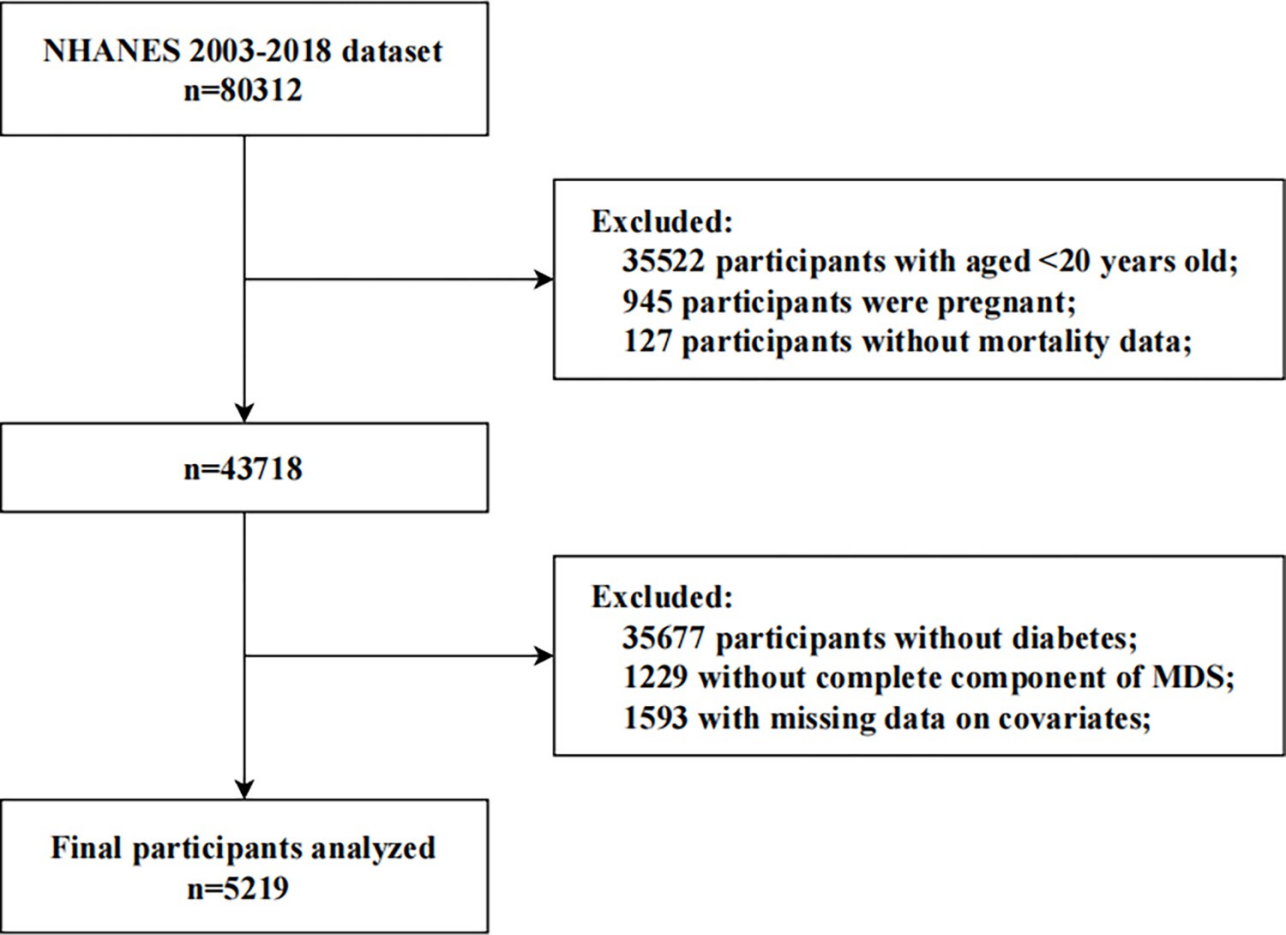
Flow diagram of the screening and enrollment of study participants. MDS, magnesium depletion score.

Our study utilized data from 80,312 participants across eight cycles of NHANES from 2003 to 2018. First, we excluded participants who were under the age of 20 (n = 35522), pregnant (n = 945), or lacking mortality data (n = 127). Subsequently, we excluded participants without diabetes (n = 35677), without complete components of MDS (n = 1229), and with missing data on covariates (n = 1593). Finally, 5,219 eligible participants were included in the analysis (Fig 1).

### Definition of MDS

We calculated the MDS score following the method established by the creators of the MDS. The score is the sum of the following four risk factors: (1) Current use of diuretics scores 1 point, (2) Current use of proton pump inhibitors (PPI) scores 1 point, (3) Heavy drinker scores 1 point, and (4) estimated glomerular filtration rate (eGFR) between 60 and <90 mL/min/1.73 m^2^ scores 1 point, while eGFR <60 mL/min/1.73 m^2^ scores 2 points [13]. Information regarding participants’ use of diuretics and PPI was obtained from the "RXQ_RX" questionnaire. Detailed information on diuretics and PPI can be found in S1 Table. The eGFR was calculated using the Chronic Kidney Disease Epidemiology Collaboration (CKD-EPI) equation from 2009. Heavy drinkers were classified as women who consume more than one drink per day on average and men who consume more than two drinks per day on average. One drink is defined as one 12-ounce beer, one 5-ounce glass of wine, or one 1.5-ounce shot of distilled spirits. Therefore, the MDS score ranges from 0 to 5 points. When MDS is treated as a categorical variable, based on previous literature, we divided MDS into lower MDS (0–1 points), middle MDS (2 points), and higher MDS (3–5 points) groups [20, 21].

### Definition of diabetes

Participants were diagnosed with diabetes if they met any of the following criteria: (1) previously diagnosed by a doctor, (2) fasting blood glucose ≥7 mmol/L, (3) glycated hemoglobin A1c (HbA1c) ≥6.5%, (4) random blood glucose ≥11.1 mmol/L, (5) 2-hour OGTT blood glucose ≥11.1 mmol/L, or (6) use of diabetes medication or insulin [22].

### Mortality outcomes

NHANES operates as a cross-sectional survey conducted in two-year cycles, with participants’ survival status data can be obtained through the National Death Index database maintained by the NCHS. The follow-up period spans from the examination date at the Mobile Examination Center (MEC) to either the date of death or December 31, 2019, whichever occurs first. Cardiovascular mortality was determined based on the International Classification of Diseases, Tenth Revision (ICD-10) codes (I00-I09, I11, I13, I20-I51) [23].

### Covariates

Based on previous literature and clinical experience, the following variables were included in this study: age, sex, race/ethnicity, educational level, family poverty income ratio (PIR), body mass index (BMI), smoking status, drinking status, hypertension, hyperlipidemia, history of cardiovascular disease (CVD), HbA1c, total cholesterol (TC), high-density cholesterol (HDL), energy intake, and magnesium intake [17, 24, 25]. Self-reported race/ethnicity was categorized as Mexican American, other Hispanic, non-Hispanic White, non-Hispanic Black, and other races. PIR was divided into three categories: ≤1.30, 1.31–3.50, and >3.50 [25]. BMI is calculated by dividing weight by the square of height. Smoking status was classified into three categories: Never smokers (defined as smoking fewer than 100 cigarettes throughout their lifetime), Current smokers (categorized as smoking more than 100 cigarettes in their lifetime and still currently smoking), and former smokers (identified as smoking more than 100 cigarettes and had subsequently quit smoking) [26]. Drinking status was categorized into five groups: never (had <12 drinks in lifetime), former (had ≥12 drinks in 1 year and did not drink last year, or did not drink last year but drank ≥12 drinks in lifetime), mild (≤1 drinks per day for females, ≤2 drinks per day for males), moderate drinker (≥2 drinks per day for females, ≥3 drinks per day for males, or binge drinking ≥2 days per month), and heavy (≥3 drinks per day for females, ≥4 drinks per day for males, or binge drinking on 5 or more days per month). Hypertension was defined as self-reported diagnosis or a measured average systolic/diastolic blood pressure of ≥140/90 mmHg. Hyperlipidemia was defined as meeting any of the following criteria: (1) use of lipid-lowering medication, (2) triglycerides (TG) ≥150 mg/dL, or (3) high cholesterol (TC ≥200 mg/dL, low-density cholesterol [LDL] ≥130 mg/dL, or HDL <40 mg/dL). Dietary energy and magnesium intake were derived from the average of two non-consecutive 24-hour dietary recalls. All variables in this study are publicly available and can be freely accessed on the NHANES website.

### Statistical analysis

Given the complex sampling design of NHANES, all analyses in this study accounted for the appropriate sample weights. The baseline characteristics of the participants were presented according to the categorical variable MDS. Continuous variables were expressed as mean (standard deviation [SD]), while categorical variables were presented as unweighted numbers (weighted percentages [%]). Continuous variables were analyzed using analysis of variance (ANOVA), while categorical variables were analyzed using the chi-square test. The Kaplan-Meier survival curves and log-rank tests were used to assess the potential differences in mortality among diabetic patients across different MDS groups. Three multivariable Cox regression models were used to examine the relationship between MDS and mortality. Model 1 was the crude model, with no adjustments for any variables. Model 2 was adjusted for age, sex, race/ethnicity, educational level, smoking status, and drinking status. Model 3 was further adjusted for BMI, PIR, hypertension, hyperlipidemia, history of CVD, HbA1c, TC, HDL, energy intake, and magnesium intake. Additionally, we used Cox regression models and likelihood ratio tests to conduct interaction and subgroup analyses based on age, sex, BMI, history of CVD, and hypertension.

To verify the stability of our results, we conducted a series of sensitivity analyses. First, we repeated the multivariable regression after excluding patients who died within the first two years of follow-up to avoid reverse causality. Second, we considered MDS as a continuous variable or reclassified it into two groups (<3 points, and ≥3 points) to further explore the association between MDS and mortality. Third, we excluded participants with missing LDL and TG data and further adjusted for TG and LDL levels in Model 3 (the fully adjusted model) to observe if the relationship between MDS and mortality changed.

All analyses were performed with R Statistical Software (https://www.R-project.org, The R Foundation) and Free Statistics software versions 1.9.2. A two-tailed P < 0.05 was considered statistically significant in all analyses.

## Results

### Characteristics of the study participants

The baseline characteristics and detailed information of the 5,219 diabetic patients are presented in Table 1. The participants in this study are representative of approximately 25.17 million diabetic patients in the United States. Based on weighted analysis, the average age of participants included in this study was 59.26 years, with 50.35% being male. After dividing MDS into three groups, there were 2,830 individuals in the lower MDS group, 1,362 individuals in the middle MDS group, and 1,027 individuals in the higher MDS group. Additionally, compared to individuals in the lower MDS group, those in the higher MDS group were more likely to be older women, non-Hispanic white, have a history of CVD and hypertension, and less likely to have hyperlipidemia. They also had lower levels of education, HbA1c, TC, dietary magnesium intake, and energy intake, along with higher HDL levels.

**Table 1:** Weighted baseline characteristics of participants in three MDS groups^a^.

Characteristics	Overall (N = 5219)	Lower MDS (N = 2830), 0–1 points	Middle MDS (N = 1362), 2 points	Higher MDS (N = 1027), 3–5 points	P value
**Age, mean (SD), years**	59.26 (13.58)	54.30 (13.22)	63.44 (11.59)	68.39 (10.27)	< 0.001
**Sex, n (%)**					0.019
Male	2681 (50.35)	1490 (52.91)	713 (49.51)	478 (43.55)	
Female	2538 (49.65)	1340 (47.09)	649 (50.49)	549 (56.45)	
**Race/ethnicity, n (%)** ^**b**^					< 0.001
Non-Hispanic White	2117 (64.69)	952 (58.24)	661 (71.91)	504 (73.77)	
Non-Hispanic Black	1270 (13.48)	657 (13.73)	337 (12.82)	276 (13.76)	
Mexican American	953 (8.98)	637 (12.04)	189 (5.34)	127 (5.03)	
Other Hispanic	480(5.61)	302 (7.12)	103 (4.00)	75 (3.39)	
Other Race^c^	399 (7.23)	282 (8.88)	72 (5.93)	45 (4.05)	
**BMI, mean (SD), kg/m** ^**2**^	33.11 (7.46)	32.87 (7.50)	33.06 (7.23)	33.94 (7.63)	0.086
**PIR, n (%)**					0.092
≤1.3	1757 (24.30)	963 (24.29)	424 (23.40)	370 (25.74)	
1.31–3.5	2150 (39.61)	1121 (37.59)	589 (41.15)	440 (43.60)	
>3.5	1312 (36.08)	746 (38.12)	349 (35.45)	217 (30.66)	
**Educational level, n (%)**					0.027
Less than high school	1655 (22.13)	905 (22.45)	408 (19.85)	342 (24.70)	
High school or equivalent	1266 (25.99)	630 (23.71)	354 (28.93)	282 (28.55)	
Above high school	2298 (51.89)	1295 (53.84)	600 (51.22)	403 (46.75)	
**Smoking status, n (%)**					< 0.001
Never	2570 (48.56)	1489 (50.66)	621 (46.82)	460 (44.68)	
Former	1819 (35.53)	822 (30.74)	553 (39.86)	444 (43.87)	
Current	830 (15.91)	519 (18.06)	188 (13.32)	123 (11.46)	
**Drinking status, n (%)**					< 0.001
Never	850 (14.12)	483 (14.68)	195 (12.35)	172 (15.16)	
Former	1597 (26.38)	817 (23.95)	442 (29.79)	338 (28.75)	
Mild	1625 (34.81)	987 (39.20)	400 (30.50)	238 (27.70)	
Moderate	534 (12.00)	228 (9.23)	158 (14.50)	148 (16.83)	
Heavy	613 (12.68)	315 (12.94)	167 (12.87)	131 (11.56)	
**History of CVD, n (%)**					< 0.001
Yes	1359 (24.76)	431 (13.98)	447 (32.02)	481 (47.44)	
No	3860 (75.24)	2399 (86.02)	915 (67.98)	546 (52.56)	
**Hyperlipidemia, n (%)**					< 0.001
Yes	642 (11.13)	394 (13.54)	161 (8.69)	87 (7.35)	
No	4577 (88.87)	2436 (86.46)	1201 (91.31)	940 (92.65)	
**Hypertension, n (%)**					< 0.001
Yes	3784 (71.38)	1727 (61.00)	1117 (79.63)	940 (91.25)	
No	1435 (28.62)	1103 (39.00)	245 (20.37)	87 (8.75)	
**HbA1c, mean (SD), %**	7.07 (1.60)	7.20 (1.73)	6.91 (1.42)	6.89 (1.40)	< 0.001
**TC, mean (SD), mg/dL**	188.79 (47.98)	194.55 (50.47)	182.99 (43.41)	179.67 (44.13)	< 0.001
**HDL, mean (SD), mg/dL**	47.71 (14.66)	47.02 (14.03)	48.46 (15.50)	48.74 (15.16)	0.034
**Magnesium intake, mean (SD), mg/day**	281.88 (115.38)	295.10 (120.08)	272.72 (110.21)	254.53 (101.20)	< 0.001
**Energy intake, mean (SD), kcal/day**	1921.42 (751.36)	2012.75 (769.83)	1857.05 (736.03)	1733.90 (666.84)	< 0.001
**Heavy drinker, n (%)**	1133 (24.31)	534 (21.61)	323 (27.27)	276 (28.20)	0.016
**Diuretics, n (%)**	1663 (29.85)	221 (7.48)	600 (42.59)	842 (80.54)	< 0.001
**PPI, n (%)**	846 (16.17)	115 (3.70)	287 (23.54)	444 (44.03)	< 0.001
**eGFR scores, n (%)**					< 0.001
0 points	2133 (42.17)	1933 (67.99)	187 (16.44)	13 (0.98)	
1 point	2011 (39.05)	897 (32.01)	836 (60.52)	278 (27.56)	
2 points	1075 (18.79)	0 (0.00)	339 (23.04)	736 (71.45)	

Abbreviations: MDS, magnesium depletion score; N, number; SD, standard deviation; BMI, body mass index; PIR, family poverty income ratio; CVD, cardiovascular disease; HbA1c, glycohemoglobin; TC, total cholesterol; HDL, high-density lipoprotein cholesterol; PPI, proton pump inhibitors; eGFR, estimated glomerular filtration rate. ^a^Data are presented as the weighted means (SD) for continuous variables or as numbers (weighted percentages, %) for categorical variables. ^b^Race/ethnicity were self-reported. ^c^Includes multiracial participants. NHANES does not provided a detailed list of all races and ethnicities.

### Kaplan‐Meier analysis

**Fig 2:**
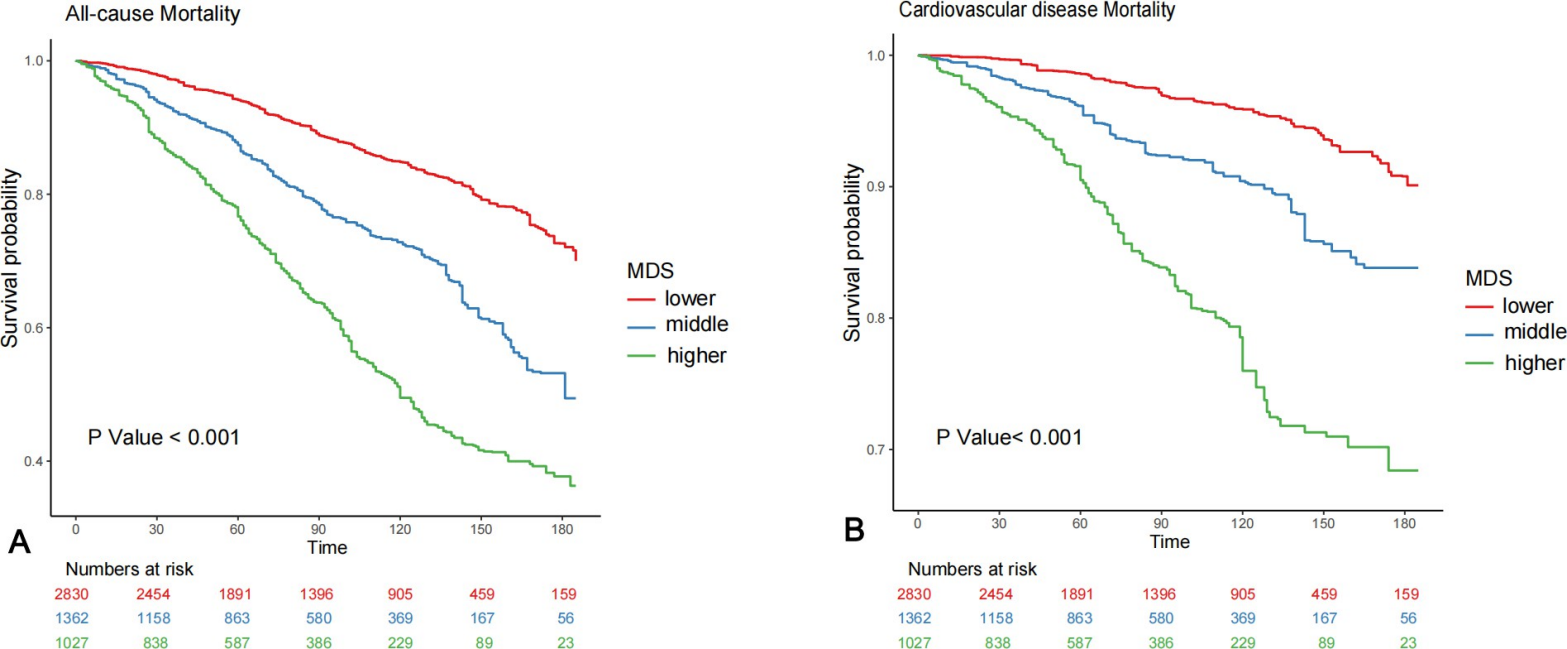
Weighted Kaplan‒Meier survival curve for all-cause mortality (A) and cardiovascular mortality (B) by MDS. MDS, magnesium depletion score.

During the median follow-up period of 81 months, there were 1,212 all-cause deaths and 348 cardiovascular deaths among the 5,219 participants in this study. The Kaplan-Meier curves showed significant differences in mortality rates among the different MDS groups (all p < 0.001), with higher MDS associated with increased all-cause (Fig 2A) and cardiovascular mortality (Fig 2B) rates compared to the lower MDS group.

### Association of MDS with all-cause and cardiovascular mortality

Table 2 presents the relationship between MDS and both all-cause mortality and cardiovascular mortality using three models from the Cox regression analysis. In Model 1 (crude model), participants in the middle and higher MDS group had a higher risk of both all-cause mortality and cardiovascular mortality compared to those in the lower MDS group. In Model 3 (the fully adjusted model), participants in the higher MDS group remained associated with an increased risk of both all-cause mortality (HR: 1.58, 95%CI: 1.20–2.08) and cardiovascular mortality (HR: 1.92, 95%CI: 1.28–2.88).

**Table 2:** The relationships between MDS and mortality in diabetes.

Characteristic	Cases/participants	Model 1^a^		Model 2^b^		Model 3^c^	
		HR (95%CI)	P value	HR (95%CI)	P value	HR (95%CI)	P value
**All-cause mortality**							
Lower MDS	404/2830	1 [Reference]		1 [Reference]		1 [Reference]	
Middle MDS	393/1362	2.11 (1.71, 2.62)	<0.001	1.30 (1.04, 1.61)	0.019	1.17 (0.93, 1.46)	0.177
Higher MDS	415/1027	3.95 (3.13, 4.98)	<0.001	1.84 (1.43, 2.37)	<0.001	1.58 (1.20, 2.08)	0.001
Trend test			<0.001		<0.001		0.001
**CVD mortality**							
Lower MDS	100/2830	1 [Reference]		1 [Reference]		1 [Reference]	
Middle MDS	110/1362	2.32 (1.51, 3.56)	<0.001	1.41 (0.90, 2.22)	0.132	1.17 (0.75, 1.82)	0.489
Higher MDS	138/1027	5.51 (3.78, 8.02)	<0.001	2.51 (1.74, 3.63)	<0.001	1.92 (1.28, 2.88)	0.001
Trend test			<0.001		<0.001		0.001

Abbreviations: MDS, magnesium depletion score; HR, Hazard ratio; CI, confidence interval; CVD, cardiovascular disease; BMI, body mass index; PIR, family poverty income ratio; HbA1c, glycohemoglobin; TC, total cholesterol; HDL, high-density lipoprotein cholesterol. ^a^Crude model. ^b^Adjusted for age, sex, race/ethnicity, educational level, smoking status, and drinking status. ^c^Adjusted for age, sex, race/ethnicity, BMI, smoking status, drinking status, educational level, PIR, hypertension, hyperlipidemia, history of CVD, HbA1c, TC, HDL, magnesium intake, and energy intake.

### Subgroup analysis

The results of the subgroup analyses are presented in Tables 3 and 4. We found that the association between MDS and all-cause mortality significantly differed between individuals aged <60 and those aged ≥60 (p for interaction = 0.015). The positive association between MDS and all-cause mortality was stronger among participants aged <60. No significant interactions were found between MDS and sex, BMI, history of CVD, or hypertension. No significant differences were found in the relationship between MDS and cardiovascular mortality across the different subgroups.

**Table 3:** Subgroup analysis of the associations between MDS and all-cause mortality among diabetes.

	Lower MDS	Middle MDS		Higher MDS		P interaction
All-cause mortality	HR (95%CI)	HR (95%CI)	P value	HR (95%CI)	P value	
**Age, years**						0.015
<60	1 [Reference]	1.38 (0.80, 2.38)	0.240	2.89 (1.56, 5.35)	<0.001	
≥60	1 [Reference]	1.06 (0.84, 1.33)	0.630	1.37 (1.04, 1.82)	0.030	
**Sex**						0.605
Male	1 [Reference]	1.21 (0.92, 1.59)	0.170	1.84 (1.24, 2.73)	0.002	
Female	1 [Reference]	1.13 (0.79, 1.62)	0.490	1.36 (0.98, 1.89)	0.070	
**BMI, kg/m** ^**2**^						0.480
<30	1 [Reference]	0.97 (0.74, 1.26)	0.800	1.13 (0.83,1.55)	0.430	
≥30	1 [Reference]	1.33 (0.95, 1.87)	0.100	2.09 (1.39, 3.16)	<0.001	
**History of CVD**						0.492
Yes	1 [Reference]	1.09 (0.80, 1.48)	0.570	1.72 (1.24, 2.39)	0.001	
NO	1 [Reference]	1.20 (0.88, 1.63)	0.250	1.38 (0.89, 2.12)	0.150	
**Hypertension**						0.764
Yes	1 [Reference]	1.16 (0.90, 1.50)	0.260	1.59 (1.17, 2.16)	0.003	
NO	1 [Reference]	1.05 (0.62, 1.75)	0.870	1.58 (0.80, 3.13)	0.190	

Abbreviations: MDS, magnesium depletion score; HR, Hazard ratio; CI, confidence interval; BMI, body mass index; PIR, family poverty income ratio; CVD, cardiovascular disease; HbA1c, glycohemoglobin; TC, total cholesterol; HDL, high-density lipoprotein cholesterol. HRs were adjusted for age, sex, race/ethnicity, BMI, smoking status, drinking status, educational level, PIR, hypertension, hyperlipidemia, history of CVD, HbA1c, TC, HDL, magnesium intake, and energy intake.

**Table 4:** Subgroup analysis of the associations between MDS and CVD mortality among diabetes.

	Lower MDS	Middle MDS		Higher MDS		P interaction
CVD mortality	HR (95%CI)	HR (95%CI)	P value	HR (95%CI)	P value	
**Age, years**						0.235
<60	1 [Reference]	1.63 (0.42, 6.26)	0.480	3.91 (1.48, 10.38)	0.010	
≥60	1 [Reference]	0.98 (0.65, 1.49)	0.940	1.57 (1.04, 2.36)	0.030	
**Sex**						0.687
Male	1 [Reference]	1.17 (0.67, 2.03)	0.570	1.88 (1.02, 3.48)	0.040	
Female	1 [Reference]	1.32 (0.66, 2.61)	0.430	1.92 (1.03, 3.58)	0.040	
**BMI, kg/m** ^**2**^						0.156
<30	1 [Reference]	0.69 (0.39, 1.20)	0.190	1.25 (0.70,2.25)	0.460	
≥30	1 [Reference]	1.68 (0.84, 3.36)	0.140	2.74 (1.49, 5.06)	0.001	
**History of CVD**						0.604
Yes	1 [Reference]	1.17 (0.69, 1.97)	0.560	2.33 (1.40, 3.86)	0.001	
NO	1 [Reference]	1.25 (0.65, 2.39)	0.500	1.35 (0.71, 2.57)	0.350	
**Hypertension**						0.465
Yes	1 [Reference]	1.26 (0.75, 2.09)	0.380	1.94 (1.21, 3.11)	0.010	
NO	1 [Reference]	0.55 (0.18, 1.67)	0.290	2.22 (0.84, 5.86)	0.110	

Abbreviations: MDS, magnesium depletion score; HR, Hazard ratio; CI, confidence interval; BMI, body mass index; PIR, family poverty income ratio; CVD, cardiovascular disease; HbA1c, glycohemoglobin; TC, total cholesterol; HDL, high-density lipoprotein cholesterol. HRs were adjusted for age, sex, race/ethnicity, BMI, smoking status, drinking status, educational level, PIR, hypertension, hyperlipidemia, history of CVD, HbA1c, TC, HDL, magnesium intake, and energy intake.

### Sensitivity analysis

First, after excluding patients who died within the first two years of follow-up, higher MDS remained significantly associated with increased all-cause and cardiovascular mortality (S2 Table). Second, when we considered MDS as a continuous variable or reclassified it into two groups (<3 points, and ≥3 points), the association between MDS and all-cause and cardiovascular mortality remained consistent with the main analysis results (S3 Table). Finally, after further excluding participants with missing TG and LDL data and additionally adjusting for TG and LDL in Model 3 (the fully adjusted model), the positive association between MDS and mortality remained unchanged (S4 Table).

## Discussion

In this large cohort study, we found that an increase in MDS is significantly correlated with a higher risk of all-cause and cardiovascular mortality in diabetic patients. In the subgroup analysis, we found that the association between MDS and all-cause mortality differed significantly across age strata (<60 years and ≥60 years). No significant interactions were found in the remaining subgroups. Three sensitivity analyses confirmed the robustness of our findings.

Serum magnesium level is a convenient and commonly used clinical indicator for assessing systemic magnesium status in clinical practice. However, serum magnesium levels do not accurately reflect systemic magnesium status [27]. Since serum magnesium makes up just 1% of the total body magnesium, with the majority stored in bones, muscles, and soft tissues, it is possible for the body to be deficient in magnesium while serum magnesium levels remain within the normal range [16]. Urine magnesium level is another clinical indicator for assessing magnesium status. However, renal excretion and reabsorption of magnesium can fluctuate easily, and urine magnesium level is not a routine test, making it neither a convenient nor effective indicator [27]. The magnesium tolerance test (MTT) is regarded as the gold standard for diagnosing systemic magnesium status. However, it requires the collection of two 24-hour urine samples, making its widespread use impractical [28]. Recently, Fan et al. developed the MDS, which considers four common risk factors influencing renal magnesium reabsorption in U.S. adults, as more than 80% of serum magnesium undergoes filtration and reabsorption in the kidneys [13]. Their research demonstrated that MDS has a higher predictive performance (AUC: 0.60, 95% CI: 0.48–0.72) for magnesium deficiency compared to serum (AUC: 0.53, 95% CI: 0.40–0.67) and urine (AUC: 0.58, 95% CI: 0.45–0.71) magnesium levels. Additionally, since the four risk factors included in MDS (current use of diuretics and PPIs, heavy alcohol consumption, and kidney disease) are easily assessable in clinical practice, MDS is a simple, practical, and effective tool for evaluating systemic magnesium status.

Earlier research has demonstrated that MDS is linked to the development of diabetes, and this association is stronger in groups with low dietary magnesium intake [17]. However, to our knowledge, no studies have explored the potential association between MDS and mortality in diabetic patients. Therefore, we designed and conducted this study, discovering that higher MDS is significantly associated with increased all-cause and cardiovascular mortality risk in diabetic patients. Previous studies have primarily focused on the association between MDS and the risk of chronic diseases such as kidney stones, COPD, and metabolic syndrome [29–31]. Currently, only three studies have explored the relationship between MDS and mortality in specific populations. The main conclusions of their studies are generally consistent with the findings of our research. Yin et al.’s study indicated that CKD patients with an MDS score >2 were significantly associated with increased all-cause and cardiovascular mortality and this association was more pronounced in groups with insufficient magnesium intake [32]. Studies by Ye et al. and Song et al. demonstrated that the positive association between MDS and both all-cause and cardiovascular mortality persisted among individuals with cardiovascular disease and hypertension [24, 25]. However, Song et al.’s study found that the association between MDS and the risk of all-cause and cardiovascular mortality in hypertensive individuals differed significantly across subgroups with and without a history of cardiovascular disease. These studies collectively indicate that MDS is significantly associated with mortality in certain populations, underscoring the importance of early monitoring and management of MDS to improve outcomes in these specific groups. Our study highlights the impact of MDS on the prognosis of diabetic patients. Timely monitoring and management of MDS may benefit diabetic patients.

Additionally, Song et al.’s study found that the association between MDS and the risk of all-cause and cardiovascular mortality in hypertensive individuals differed significantly across subgroups with and without a history of cardiovascular disease [25]. Our subgroup analysis indicated that there was no significant interaction between MDS and a history of cardiovascular disease. However, the association between MDS and all-cause mortality in diabetic patients showed significant differences across age subgroups. The association between higher MDS and increased risk of all-cause mortality was stronger in individuals aged <60 years. Among individuals aged <60 years, participants in the higher MDS group had an all-cause mortality rate 2.89 times higher than those in the lower MDS group, whereas among individuals aged ≥60 years, participants in the higher MDS group had an all-cause mortality rate 1.37 times higher than those in the lower MDS group. A similar difference is observed in the association between MDS and cardiovascular mortality, where this trend is evident even though the interaction is not statistically significant. Among individuals aged <60 years, individuals in the higher MDS group had a cardiovascular mortality rate 3.91 times higher than those in the lower MDS group, whereas among individuals aged ≥60 years, individuals in the higher MDS group had a cardiovascular mortality rate 1.57 times higher than those in the lower MDS group. This difference may be attributed to younger diabetic patients generally having higher BMI and HbA1c levels compared to older diabetic patients [33]. The increase in obesity and poorer blood glucose control can significantly exacerbate inflammation in the body, potentially amplifying the impact of magnesium deficiency on the prognosis of diabetic patients [34, 35]. Additionally, previous studies have shown that obesity can affect magnesium metabolism, exacerbating magnesium deficiency and potentially increasing adverse outcomes in diabetic patients [36, 37]. Therefore, for diabetic patients aged <60, it is crucial to timely monitor and manage MDS and optimize magnesium nutritional status to improve future outcomes.

Our research findings hold significant clinical implications. Given that MDS exhibits superior predictive performance for systemic magnesium deficiency compared to serum and urinary magnesium levels, we utilize a comprehensive evaluation of magnesium deficiency in diabetic patients by integrating these three clinical indicators in clinical practice. Additionally, our research findings suggest that diabetic patients should monitor and control MDS alongside routine blood glucose assessments to improve their adverse prognoses. Young and middle-aged diabetic patients have a greater necessity to control MDS, as their mortality risk is significantly higher than that of diabetic patients over 60 years of age. Optimizing magnesium nutritional status and supplementing with magnesium may benefit diabetic patients.

Compared to past studies, our study has several key strengths. First, this study benefits from a large sample size, providing sufficient data to explore the potential association between MDS and mortality in diabetic patients. Second, our study accounted for the complex sample weighting design of NHANES, making our findings more representative of adult diabetic patients in the United States. Third, our findings remained robust across three sensitivity analyses.

However, this study has some limitations. First, as an observational study, this research cannot establish a causal relationship between MDS and mortality in diabetic patients. Future studies with more rigorous designs are needed to explore this further. Second, since the study population is based on the U.S. population, the findings may not be generalizable to other regions and ethnicities. Thirdly, there are other medications, besides diuretics and PPIs, that can affect magnesium levels. However, diuretics and PPIs are the most commonly used medications, and these two can represent the majority of the general population [14]. Finally, although we controlled for as many confounding factors as possible, residual confounding factors may still influence the final analysis results. However, our sensitivity analyses confirmed the robustness of our findings.

## Conclusion

In conclusion, this study demonstrates that an increase in MDS is significantly correlated with a higher risk of all-cause and cardiovascular mortality in diabetic patients. The risk of all-cause mortality is significantly higher in diabetic patients aged <60 compared to those aged ≥60. Therefore, early monitoring and management of MDS, as well as optimizing magnesium nutritional status, may benefit the prognosis of diabetic patients.

## Supporting information

S1 Table Detailed information on diuretics and PPI. (DOCX)

S2 Table Weighted multivariable Cox regression analysis of MDS and mortality in diabetic patients after excluding those who died within 2 years of follow-up. (DOCX)

S3 Table Weighted multivariable Cox regression analysis of MDS and mortality in diabetic patients when MDS is treated as a continuous variable or reclassified into two groups (<3 points, ≥3 points). (DOCX)

S4 Table Weighted multivariable Cox regression analysis of MDS and mortality in diabetic patients after excluding participants with missing TG and LDL data. (DOCX)

S1 Data (CSV)
