# Supplementary material for: Higher magnesium depletion score increases the risk of all‑cause and cardiovascular mortality in US adults with diabetes
Source: PLoS One. 2025 Jan 20;20(1):e0314298. doi: 10.1371/journal.pone.0314298 (PMC11745414; doi:10.1371/journal.pone.0314298)
Supplement: S1 Table — (DOCX) [file pone.0314298.s001.docx]

**S1 Table** Detailed Information on Diuretics and PPI

| Diuretics |  |  | PPI |  |
| --- | --- | --- | --- | --- |
| Generic drug code | **Generic drug name** |  | **Generic drug code** | **Generic drug name** |
| c00049 | DIURETICS - UNSPECIFIED |  | d00325 | OMEPRAZOLE |
| c00156 | THIAZIDE AND THIAZIDE-LIKE DIURETICS - UNSPECIFIED |  | d03828 | LANSOPRAZOLE |
| d00070 | FUROSEMIDE |  | d04448 | RABEPRAZOLE |
| d00161 | ACETAZOLAMIDE |  | d04514 | PANTOPRAZOLE |
| d00169 | AMILORIDE |  | d04749 | ESOMEPRAZOLE |
| d00179 | BUMETANIDE |  | d05770 | OMEPRAZOLE; SODIUM BICARBONATE |
| d00190 | CHLOROTHIAZIDE |  | d07395 | DEXLANSOPRAZOLE |
| d00192 | CHLORTHALIDONE |  | d04913 | LANSOPRAZOLE; NAPROXEN |
| d00253 | HYDROCHLOROTHIAZIDE |  | d07631 | ESOMEPRAZOLE; NAPROXEN |
| d00260 | INDAPAMIDE |  | d04272 | AMOXICILLIN; CLARITHROMYCIN; LANSOPRAZOLE |
| d00299 | METOLAZONE |  | d05399 | AMOXICILLIN; CLARITHROMYCIN; OMEPRAZOLE |
| d00373 | SPIRONOLACTONE |  |  |  |
| d00396 | TRIAMTERENE |  |  |  |
| d00639 | DICHLORPHENAMIDE |  |  |  |
| d00640 | METHAZOLAMIDE |  |  |  |
| d00641 | BENDROFLUMETHIAZIDE |  |  |  |
| d00643 | METHYCLOTHIAZIDE |  |  |  |
| d00645 | HYDROFLUMETHIAZIDE |  |  |  |
| d00646 | TRICHLORMETHIAZIDE |  |  |  |
| d00649 | ETHACRYNIC ACID |  |  |  |
| d03189 | TORSEMIDE |  |  |  |
| d04815 | EPLERENONE |  |  |  |
| d03052 | HYDROCHLOROTHIAZIDE; TRIAMTERENE |  |  |  |
| d03193 | AMILORIDE; HYDROCHLOROTHIAZIDE |  |  |  |
| d03247 | HYDROCHLOROTHIAZIDE; SPIRONOLACTONE |  |  |  |
| d03257 | HYDRALAZINE; HYDROCHLOROTHIAZIDE |  |  |  |
| d03830 | HYDROCHLOROTHIAZIDE; LOSARTAN |  |  |  |
| d04245 | HYDROCHLOROTHIAZIDE; IRBESARTAN |  |  |  |
| d04293 | HYDROCHLOROTHIAZIDE; VALSARTAN |  |  |  |
| d04711 | CANDESARTAN; HYDROCHLOROTHIAZIDE |  |  |  |
| d04737 | HYDROCHLOROTHIAZIDE; TELMISARTAN |  |  |  |
| d04837 | EPROSARTAN; HYDROCHLOROTHIAZIDE |  |  |  |
| d04878 | HYDROCHLOROTHIAZIDE; OLMESARTAN |  |  |  |
| d07077 | ALISKIREN; HYDROCHLOROTHIAZIDE |  |  |  |
| d07818 | AZILSARTAN; CHLORTHALIDONE |  |  |  |
| a11077 | HYDROCHLOROTHIAZIDE; LABETALOL |  |  |  |
| d03248 | POLYTHIAZIDE; RESERPINE |  |  |  |
| d03249 | CHLORTHALIDONE; RESERPINE |  |  |  |
| d03250 | CHLOROTHIAZIDE; RESERPINE |  |  |  |
| d03251 | HYDROCHLOROTHIAZIDE; RESERPINE |  |  |  |
| d03252 | HYDROFLUMETHIAZIDE; RESERPINE |  |  |  |
| d03253 | METHYCLOTHIAZIDE; RESERPINE |  |  |  |
| d03256 | HYDRALAZINE; HYDROCHLOROTHIAZIDE; RESERPINE |  |  |  |
| d03258 | ATENOLOL; CHLORTHALIDONE |  |  |  |
| d03259 | BENDROFLUMETHIAZIDE; NADOLOL |  |  |  |
| d03260 | HYDROCHLOROTHIAZIDE; TIMOLOL |  |  |  |
| d03263 | HYDROCHLOROTHIAZIDE; METHYLDOPA |  |  |  |
| d03264 | HYDROCHLOROTHIAZIDE; METOPROLOL |  |  |  |
| d03265 | BENAZEPRIL; HYDROCHLOROTHIAZIDE |  |  |  |
| d03266 | HYDROCHLOROTHIAZIDE; LISINOPRIL |  |  |  |
| d03267 | CHLORTHALIDONE; CLONIDINE |  |  |  |
| d03268 | POLYTHIAZIDE; PRAZOSIN |  |  |  |
| d03564 | DESERPIDINE; METHYCLOTHIAZIDE |  |  |  |
| d03566 | CAPTOPRIL; HYDROCHLOROTHIAZIDE |  |  |  |
| d03740 | ENALAPRIL; HYDROCHLOROTHIAZIDE |  |  |  |
| d03744 | BISOPROLOL; HYDROCHLOROTHIAZIDE |  |  |  |
| d03778 | CHLOROTHIAZIDE; METHYLDOPA |  |  |  |
| d04141 | HYDROCHLOROTHIAZIDE; MOEXIPRIL |  |  |  |
| d04509 | HYDROCHLOROTHIAZIDE; QUINAPRIL |  |  |  |
| d04539 | FOSINOPRIL; HYDROCHLOROTHIAZIDE |  |  |  |
| d07440 | AMLODIPINE; HYDROCHLOROTHIAZIDE; VALSARTAN |  |  |  |
| d07668 | AMLODIPINE; HYDROCHLOROTHIAZIDE; OLMESARTAN |  |  |  |
| d03261 | HYDROCHLOROTHIAZIDE; PROPRANOLOL |  |  |  |

PPI, proton pump inhibitors. The selection of diuretics and PPIs was based on the therapeutic classification codes from the "RXQ_DRUG" file. The first-level and second-level category IDs for diuretics are 40 and 49, respectively. For PPIs, the first-level and second-level category IDs are 87 and 272, respectively.
