# Supplementary material for: Higher magnesium depletion score increases the risk of all‑cause and cardiovascular mortality in US adults with diabetes
Source: PLoS One. 2025 Jan 20;20(1):e0314298. doi: 10.1371/journal.pone.0314298 (PMC11745414; doi:10.1371/journal.pone.0314298)
Supplement: S2 Table — (DOCX) [file pone.0314298.s002.docx]

**S2 Table** Weighted multivariable Cox regression analysis of MDS and mortality in diabetic patients after excluding those who died within 2 years of follow-up

|  | **Cases/participants** | **Model 1^a^** |  |  | **Model 2^b^** |  |  | **Model 3^c^** |  |
| --- | --- | --- | --- | --- | --- | --- | --- | --- | --- |
|  |  | **HR (95%CI)** | **P value** |  | **HR (95%CI)** | **P value** |  | **HR (95%CI)** | **P value** |
| **All-cause mortality** |  |  |  |  |  |  |  |  |  |
| Lower MDS | 356/2582 | 1 [Reference] |  |  | 1 [Reference] |  |  | 1 [Reference] |  |
| Middle MDS | 334/1203 | 2.03 (1.62, 2.53) | <0.001 |  | 1.24 (0.98, 1.55) | 0.068 |  | 1.11 (0.87, 1.41) | 0.387 |
| Higher MDS | 337/885 | 3.77 (2.94, 4.84) | <0.001 |  | 1.74 (1.31, 2.31) | <0.001 |  | 1.52 (1.13, 2.04) | 0.006 |
| Trend test |  |  | <0.001 |  |  | <0.001 |  |  | 0.007 |
| **CVD mortality** |  |  |  |  |  |  |  |  |  |
| Lower MDS | 94/2582 | 1 [Reference] |  |  | 1 [Reference] |  |  | 1 [Reference] |  |
| Middle MDS | 94/1203 | 2.12 (1.35, 3.33) | 0.001 |  | 1.24 (0.78, 1.97) | 0.355 |  | 1.01 (0.63, 1.63) | 0.955 |
| Higher MDS | 107/885 | 4.79 (3.17, 7.23) | <0.001 |  | 2.08 (1.40, 3.10) | <0.001 |  | 1.55 (1.02, 2.36) | 0.038 |
| Trend test |  |  | <0.001 |  |  | <0.001 |  |  | 0.037 |

Abbreviations: MDS, magnesium depletion score; HR, Hazard ratio; CI, confidence interval; CVD, cardiovascular disease; BMI, body mass index; PIR, family poverty income ratio; HbA1c, glycohemoglobin; TC, total cholesterol; HDL, high-density lipoprotein cholesterol. ^a^Crude model. ^b^Adjusted for age, sex, race/ethnicity, educational level, smoking status, and drinking status. ^c^Adjusted for age, sex, race/ethnicity, BMI, smoking status, drinking status, educational level, PIR, hypertension, hyperlipidemia, history of CVD, HbA1c, TC, HDL, magnesium intake, and energy intake.
