# Supplementary material for: Higher magnesium depletion score increases the risk of all‑cause and cardiovascular mortality in US adults with diabetes
Source: PLoS One. 2025 Jan 20;20(1):e0314298. doi: 10.1371/journal.pone.0314298 (PMC11745414; doi:10.1371/journal.pone.0314298)
Supplement: S3 Table — (DOCX) [file pone.0314298.s003.docx]

**S3 Table** Weighted multivariable Cox regression analysis of MDS and mortality in diabetic patients when MDS is treated as a continuous variable or reclassified into two groups (<3 points, ≥3 points)

|  | **Cases/participants** | **Model 1^a^** |  |  | **Model 2^b^** |  |  | **Model 3^c^** |  |
| --- | --- | --- | --- | --- | --- | --- | --- | --- | --- |
|  |  | **HR (95%CI)** | **P value** |  | **HR (95%CI)** | **P value** |  | **HR (95%CI)** | **P value** |
| **All-cause mortality** |  |  |  |  |  |  |  |  |  |
| MDS | 1212/5219 | 1.66 (1.51, 1.81) | <0.001 |  | 1.28 (1.14, 1.43) | <0.001 |  | 1.21 (1.07, 1.37) | 0.002 |
| MDS category |  |  |  |  |  |  |  |  |  |
| MDS<3 | 797/4192 | 1 [Reference] |  |  | 1 [Reference] |  |  | 1 [Reference] |  |
| MDS≥3 | 415/1027 | 2.94 (2.39, 3.61) | <0.001 |  | 1.59 (1.29, 1.97) | <0.001 |  | 1.44 (1.16, 1.80) | 0.001 |
| **CVD mortality** |  |  |  |  |  |  |  |  |  |
| MDS | 348/5219 | 1.82 (1.56, 2.12) | <0.001 |  | 1.41 (1.20, 1.65) | <0.001 |  | 1.28 (1.07, 1.55) | 0.009 |
| MDS category |  |  |  |  |  |  |  |  |  |
| MDS<3 | 210/4192 | 1 [Reference] |  |  | 1 [Reference] |  |  | 1 [Reference] |  |
| MDS≥3 | 138/1027 | 3.91 (2.81, 5.43) | <0.001 |  | 2.07 (1.53, 2.81) | <0.001 |  | 1.75 (1.26, 2.43) | <0.001 |

Abbreviations: MDS, magnesium depletion score; HR, Hazard ratio; CI, confidence interval; CVD, cardiovascular disease; BMI, body mass index; PIR, family poverty income ratio; HbA1c, glycohemoglobin; TC, total cholesterol; HDL, high-density lipoprotein cholesterol. ^a^Crude model. ^b^Adjusted for age, sex, race/ethnicity, educational level, smoking status, and drinking status. ^c^Adjusted for age, sex, race/ethnicity, BMI, smoking status, drinking status, educational level, PIR, hypertension, hyperlipidemia, history of CVD, HbA1c, TC, HDL, magnesium intake, and energy intake.
