# Supplementary material for: Higher magnesium depletion score increases the risk of all‑cause and cardiovascular mortality in US adults with diabetes
Source: PLoS One. 2025 Jan 20;20(1):e0314298. doi: 10.1371/journal.pone.0314298 (PMC11745414; doi:10.1371/journal.pone.0314298)
Supplement: S4 Table — (DOCX) [file pone.0314298.s004.docx]

**S4 Table** Weighted multivariable Cox regression analysis of MDS and mortality in diabetic patients after excluding participants with missing TG and LDL data

|  | **Cases/participants** | **Model 1^a^** |  |  | **Model 2^b^** |  |  | **Model 4^c^** |  |
| --- | --- | --- | --- | --- | --- | --- | --- | --- | --- |
|  |  | **HR (95%CI)** | **P value** |  | **HR (95%CI)** | **P value** |  | **HR (95%CI)** | **P value** |
| **All-cause mortality** |  |  |  |  |  |  |  |  |  |
| Lower MDS | 210/1463 | 1 [Reference] |  |  | 1 [Reference] |  |  | 1 [Reference] |  |
| Middle MDS | 223/730 | 2.75 (2.10, 3.61) | <0.001 |  | 1.65 (1.26, 2.16) | <0.001 |  | 1.46 (1.10, 1.95) | 0.009 |
| Higher MDS | 213/525 | 4.31 (3.34, 5.57) | <0.001 |  | 1.84 (1.37, 2.47) | <0.001 |  | 1.67 (1.22, 2.27) | 0.001 |
| Trend test |  |  | <0.001 |  |  | <0.001 |  |  | 0.001 |
| **CVD mortality** |  |  |  |  |  |  |  |  |  |
| Lower MDS | 53/1463 | 1 [Reference] |  |  | 1 [Reference] |  |  | 1 [Reference] |  |
| Middle MDS | 60/730 | 2.64 (1.59, 4.39) | <0.001 |  | 1.55 (0.89, 2.72) | 0.124 |  | 1.39 (0.80, 2.43) | 0.246 |
| Higher MDS | 75/525 | 6.69 (4.30, 10.41) | <0.001 |  | 2.91 (1.79, 4.72) | <0.001 |  | 2.48 (1.44, 4.26) | <0.001 |
| Trend test |  |  | <0.001 |  |  | <0.001 |  |  | 0.001 |

Abbreviations: MDS, magnesium depletion score; TG, triglycerides; LDL, low-density cholesterol; HR, Hazard ratio; CI, confidence interval; CVD, cardiovascular disease; BMI, body mass index; PIR, family poverty income ratio; HbA1c, glycohemoglobin; TC, total cholesterol; HDL, high-density lipoprotein cholesterol. ^a^Crude model. ^b^Adjusted for age, sex, race/ethnicity, educational level, smoking status, and drinking status. ^c^Adjusted for age, sex, race/ethnicity, BMI, smoking status, drinking status, educational level, PIR, hypertension, hyperlipidemia, history of CVD, HbA1c, TC, HDL, TG, LDL, magnesium intake, and energy intake.
